# Interaction of *Varroa destructor* and Sublethal Clothianidin Doses during the Larval Stage on Subsequent Adult Honey Bee (*Apis mellifera* L.) Health, Cellular Immunity, Deformed Wing Virus Levels and Differential Gene Expression

**DOI:** 10.3390/microorganisms8060858

**Published:** 2020-06-06

**Authors:** Nuria Morfin, Paul H. Goodwin, Ernesto Guzman-Novoa

**Affiliations:** School of Environmental Sciences, University of Guelph, Guelph, ON N1G 2W1, Canada; pgoodwin@uoguelph.ca (P.H.G.); eguzman@uoguelph.ca (E.G.-N.)

**Keywords:** honeybees, *Varroa destructor*, deformed wing virus, neonicotinoid insecticides, clothianidin, interaction, gene expression

## Abstract

Honeybees (*Apis mellifera* L.) are exposed to many parasites, but little is known about interactions with abiotic stressors on their health, particularly when affected as larvae. Larvae were exposed singly and in combination to the parasitic mite *Varroa destructor* and three sublethal doses of the neonicotinoid insecticide clothianidin to evaluate their effects on survivorship, weight, haemocyte counts, deformed wing virus (DWV) levels and gene expression of the adult bees that subsequently developed. Clothianidin significantly reduced bee weight at the highest dose and was associated with an increase in haemocyte counts at the lowest dose, whereas *V. destructor* parasitism increased DWV levels, reduced bee emergence, lowered weight and reduced haemocyte counts. An interaction between the two stressors was observed for weight at emergence. Among the differentially expressed genes (DEGs), *V. destructor* infestation resulted in broader down-regulatory effects related to immunity that was often shared with the combined stressors, while clothianidin resulted in a broader up-regulatory effect more related to central metabolic pathways that was often shared with the combined stressors. Parasites and abiotic stressors can have complex interactions, including additive effects on reduced weight, number of up-regulated DEGs and biological pathways associated with metabolism.

## 1. Introduction

High mortality rates of honeybee (*Apis mellifera* L.) colonies have been a major concern for the beekeeping industry in recent years in North America and parts of Europe [1,2]. Several factors have been linked to high bee mortality, including the ectoparasitic mite *Varroa destructor*, viral infections (e.g., deformed wing virus; DWV), and exposure to neonicotinoid insecticides [3,4]. However, honeybees are exposed to multiple stressors that may interact and impact honey bee health and consequently increase colony mortality.

Neonicotinoids are systemic organic insecticides composed of active nicotine-like molecules, which act as nicotinic acetylcholine receptor (nAChRs) agonists at the synaptic membranes in the central nervous system of insects, causing a neurotoxic effect [5]. Honey bees can come into contact with neonicotinoid insecticides when collecting contaminated nectar or pollen from seed-treated crops [6,7,8]. Contaminated pollen can be stored as bee bread in combs and used by the bees to feed larvae [9]. Thus, it is not uncommon that honey bees are exposed to sublethal doses of neonicotinoids and other insecticides for a prolonged period through a continued consumption of contaminated food. Although most studies conducted thus far have focused on the effect of neonicotinoids on adult bees, neonicotinoids also affect bees during the larval stage. For example, exposing honey bee larvae to imidacloprid resulted in higher expression of phenol oxidase and higher levels of DWV [10], and exposing colonies of *Bombus terrestris* or larvae of *Osmia lignaria* and *Megachile rotundata* to food contaminated with imidacloprid resulted in decreased brood production and a longer period for larval development [11,12]. Moreover, sublethal exposure to clothianidin during the larval stage may result in a decrease in hygienic and foraging behavior activities when those larvae become adult honey bees [13]. At a colony level, neonicotinoids have been reported to reduce colony performance, queen fecundity and hygienic behavior [14,15]. However, a few studies failed to detect detrimental effects of sublethal doses of neonicotinoids at the colony level when measuring mortality, feeding activity, comb production, breeding performance, colony vitality, worker longevity and brood development [16,17].

At the molecular level, neonicotinoids can affect a variety of genes of honeybees with different functions, such as immunity, detoxification and neural related genes [18,19,20]. Because neonicotinoids act as neurotoxins, it is not surprising that acetylcholinesterase (AChE) levels increase in bees exposed to corn fields treated with neonicotinoids [21]. Further, clothianidin, imidacloprid and thiamethoxam increased nAChRα expression when bees were treated in the laboratory after 72 h of treatment [18]. Cytochrome P450 enzyme expression level, which is associated with detoxification, has also been shown to be increased when bees are exposed to neonicotinoids [19]. The expression of immune-related genes can be suppressed as well by neonicotinoids, possibly promoting the establishment and replication viruses [18,22].

Honeybee immune defense mechanisms can be divided into cellular and humoral immunity, both being triggered by the presence of pathogens [23,24]. Humoral immunity in honey bees is mediated by three main pathways, Toll, Imd and Jak/STAT, whose activation culminates in the expression of antimicrobial peptides (AMPs) or the activation of other defense mechanisms, such as antiviral responses [25]. Cellular immunity is mediated by haemocytes, which are produced in the lymphatic glands of insects, and are stored under their cuticle and in other organs [26]. When haemocytes are needed to heal a wound or to attack a pathogen, they are released into the haemolymph [27]. Thus, haemocyte quantification has often been used as an indicator of immunocompetence in insects like honeybees [28,29].

*Varroa destructor* is an ectoparasitic mite of adult bees and larvae that has been highly associated with honeybee colony mortality [30,31]. Bees parasitized during the larval stage show weight reduction at emergence, decreased protein concentration and lower haemocyte counts [32,33,34]. Further, *V. destructor* has an immunosuppressive effect on its host [35,36,37,38] and acts as vector of viruses that infect honeybees, such as the deformed wing virus (DWV) and others [39,40]. Moreover, *V. destructor* and DWV have a mutualistic relationship in which the mite transmits high titers of DWV into the bee and the virus immunosuppresses the host, allowing for greater mite fecundity [41].

Studies assessing potential combinatorial effects of neonicotinoids and pathogens on honey bee health have reported contradictory results with negative effects on survival, energetic stress, flight capacity, *Nosema* spp. reproduction, and grooming behavior, but no effects on detoxification systems, haemocyte counts and phenol oxidase activity [42,43,44,45,46,47]. These differences may be due to the type of neonicotinoid, doses tested, methods of delivery, time of exposure, developmental stage of the bees, or type of pathogen interacting with the insecticide. Thus, more information on the combined effects of abiotic and biotic stressors, such as neonicotinoid insecticides and *V. destructor*, is necessary to better understand their potential combinatorial effects on the health, immunity and gene expression of one of the most important pollinators, honey bees.

In this study, the effect of chronic exposure to sublethal doses of clothianidin during the larval stage, in the presence or absence of *V. destructor*, was examined in newly emerged bees. Variables analysed included weight, haemocyte counts, DWV levels and the proportion of emerged bees. Further, analysis of total gene expression and associated biological pathways using RNAseq and the Kyoto Encyclopedia of Genes and Genomes (KEGG) was performed to gain an understanding of the breadth of the stressor effect on individual bees and the consequent impacts on their health.

## 2. Materials and Methods

### 2.1. Ethical Statement

The study was conducted under the supervision of researchers of the Honey Bee Research Centre, University of Guelph in Guelph, ON, Canada. Beekeeping practices were performed in compliance with the Ontario Ministry of Agriculture, Food and Rural Affairs (OMAFRA) bio-safety regulations. No permits were required to conduct the study.

### 2.2. Source of Honeybees and Varroa destructor

Honeybees were collected from colonies of the Buckfast strain kept at the Honey Bee Research Centre, University of Guelph, ON, Canada. The queens that provided the larvae and workers were mated under controlled conditions in isolation at Thorah Island, Simcoe, ON, Canada, to guarantee the purity and uniformity of the Buckfast strain. The colonies were not exposed to pesticides at any time during the experiment and were uniform in size and food stores. Female *V. destructor* mites were collected from highly infested colonies as per Arechavaleta-Velasco and Guzman-Novoa [48] and placed in a Petri dish and immediately used for the experiments.

### 2.3. Working Clothianidin Dilutions

The amount of clothianidin used in the experiments was estimated based on the concentration of clothianidin in maize pollen of plants from insecticide-treated seeds (0.0074 to 3.9 ng of clothianidin/mg of pollen [49]) and the total consumption of pollen by a honey bee during the larval stage (1.52–2.04 mg [50]). From this, the estimated amount of clothianidin that a honey bee larva could potentially consume from maize pollen throughout its development ranges from 0.011 to 7.95 ng. Therefore, three doses were used—0.4, 2.0 and 4.0 ng of clothianidin—which are within the range of realistic exposure to clothianidin through contaminated pollen by a honey bee larva. To prepare the working dilutions, 10 mg of clothianidin (Sigma-Aldrich, Oakville, ON, Canada) was diluted in 100 mL of H_2_O, which was then serially diluted to obtain 100, 500 and 1000 ng/mL clothianidin dilutions.

### 2.4. Exposure to Clothianidin and/or V. destructor

Larvae of the same age were obtained as per Morfin et al. [13]. Briefly, each of four sister queens was placed inside a modified hive which had two wire mesh walls (queen-excluder size) at the center of the brood chamber, into which a drawn empty comb was placed along with the queen. The confined queen was allowed to lay eggs on the comb for 24 h, and then the queen was removed to prevent more eggs being laid on the comb. One hundred larvae that hatched from the eggs of the four combs were used for the treatments as follows.

One hundred 4-day-old larvae (100 larvae replicated in four colonies) were treated as per Morfin et al. [13] with one of the four following treatments. Each larva was treated for three consecutive days with 1.33 µL of ds H_2_O containing 0 ng of clothianidin (control) or 0.13, 0.67 or 1.33 ng of clothianidin, using a 2 µL pipette (Eppendorf, Mississauga, ON, Canada). Thus, after three days of treatment, each larva would have received 0, 0.4, 2.0 or 4.0 ng of clothianidin. The 400 larvae (100 per clothianidin dose) were treated inside their comb, and the sections of combs containing treated larvae were identified by marking the frames at different heights using water-based, non-toxic markers (UniPOSCA, Mitsubishi Pencil Company, Tokyo, Japan). The frames with the treated larvae were returned to their respective colonies to continue their normal development.

Fifty of the 100 larvae treated with one of the four clothianidin doses were artificially infested with one *V. destructor* mite as per Hamiduzzaman et al. [51] one day after the cells were capped. Briefly, each capped cell was partially opened by making an incision 1.5–2.0 mm in length on its side, using a single-edged blade (GEM, West Chester, PA, USA). One mite was taken from a Petri dish and placed inside the opened cell with a fine paint brush. The viability of the mite was confirmed by probing it for movement before its introduction. After introducing the mite, the cell was resealed by lightly brushing melted beeswax on the slit. After introducing *V. destructor* mites into experimental cells, the frames were reintroduced into their respective colonies to continue their development for 10 more days.

### 2.5. Effect of Clothianidin and/or V. destructor on Bee Emergence and Weight

The frames with the treated brood were retrieved from their colonies, and the 50 cells of each treatment on each comb were covered with a 3 mm wire mesh push-in cage (11.5 × 7.5 × 1.5 cm with 2.5 mm screen), which was embedded into the comb to contain the bees that would emerge from the cells. Then, the frame was placed inside a screened emerging cage (50.3 × 7.3 × 25.2 cm) in an incubator (35 °C, 60% RH). The number of bees that emerged or did not emerge from each of the treatments were counted. The emerged bees were held by the wings and placed on a disposable polystyrene dish (812 × 812 mm; Fisherbrand, Mississauga, ON, Canada) to weigh them on an analytical balance (Denver Instrument, Bohemia, NY, USA). Three repetitions of this experiment were conducted.

### 2.6. Effect of Clothianidin and/or V. destructor on Cellular Immunity

After a bee was weighed, 4 µL of haemolymph was obtained by piercing the intersegmental membrane of dorsal tergites using a # 7 entomological pin and a 10 µL micropipette [38]. After taking the haemolymph sample, the bee was stored at −70 °C for gene analyses. The sample of haemolymph from each bee was spread over a microscope slide. The haemolymph smear was fixed using 10 mL 95% methanol and stained using the Hema 3 (Fisher Health Care PROTOCOL, Mississauga, ON, Canada). After staining, the haemocytes were counted using a microscope (Olympus BX41, Richmond Hill, ON, Canada) at 400× with a 10 × 10 mm ocular reticle (Olympus, Richmond Hill, ON, Canada), and the number of haemocytes per µL of haemolymph calculated as per Koleoglu et al. [38].

### 2.7. RNA Extraction

Total RNA was extracted from six to eight newly emerged bees from each biological repetition using TRIzol Reagent (Fisher Scientific, Mississauga, ON, Canada) following the manufacturer’s instructions. The quality and concentration of the RNA were measured by determining the nucleic acid absorbance ratio (260/280 nm) using a spectrophotometer (NanodropLite, Thermo Scientific, Mississauga, ON, Canada). Values between 1.8–2.0 for 260/280 nm and values between 2.0–2.2 for 260/230 nm were considered acceptable for purity, indicating no significant presence of contaminants, such as phenol or proteins. The RNA was used for DWV quantification and RNA sequencing (RNAseq).

### 2.8. cDNA Synthesis and DWV Quantification

cDNA was prepared using a RevertAid H Minus First Strand cDNA Synthesis Kit (Fermentas, Burlington ON, Canada) following the manufacturer’s instructions using 2000 ng of RNA for each sample. The cDNA was stored at −20 °C until used for DWV quantification.

Primers specific for the DWV helicase [22] were used to calculate the number of DWV genome copies (DWVgc) in a BioRad CFX96 thermocycler (Bio-Rad Laboratories, Mississauga, ON, Canada). The protocol consisted of one cycle at 48 °C for 15 min, one cycle at 95 °C for 10 min, 40 cycles at 95 °C for 15 s and 60 °C for 60 s, followed by one cycle at 68 °C for 7 min [22]. The reaction volume was 25 µL, containing 2 µL of template, 3 µL of 200 nM primers,12.5 µL of a Maxima SYBR Green/ROX qRT-PCR Master Mix (2×) (Thermo Scientific, Mississauga, ON, Canada) and 7.5 µL of nuclease-free H_2_O per sample. Nuclease-free H_2_O was included instead of cDNA as a negative control, and a positive control from previously identified DWV positive samples were included in each qRT-PCR run. Synthetic gene fragments of 300 bp (gBlock, Integrated DNA Technologies, Carolville, IA, USA), which included the sequence of the forward primer, amplicon and reverse primer, were used to produce calibration curves to convert Ct values to DWVgc. The lyophilized synthetic gene fragment was diluted with nuclease-free H_2_0 to obtain 10 ng/µL that was used to make serial dilutions from 10^9^ to 10^1^ copies. Using a plot of Ct values versus DWV copy number (log10), a linear equation was used to calculate the DWV genome copy numbers for the samples of interest.

### 2.9. RNA Sequencing (RNAseq)

RNA from bees exposed to 0 ng of clothianidin (non-treated control), 1.33 ng of clothianidin alone, parasitized by *V. destructor* alone, or 1.33 ng of clothianidin plus *V. destructor* were sent for RNA sequencing (RNAseq) at Génome Québec Innovation Centre (Montreal, QC, Canada). A volume of 15 µL of the RNA from each of the three biological replicates per treatment was pooled to obtain an equivalent of 24 bees, which was used for RNAseq analysis. The RNA Library preparation for Illumina sequencing was performed using the NEB kit (New England Biolabs, Ipswich, MA, USA) and the KAPA kit (Roche, Mississauga, ON, Canada) according to the manufacturer’s instructions. Sequencing was performed as 125 bp, paired-end reads in a single lane using a HiSeq2500 v. 4 (Illumina, San Diego, CA, USA).

### 2.10. Statistical Analyses and Bioinformatics

The data on proportion of bees that emerged, weight at emergence, haemocyte counts, and DWV levels were subjected to Shapiro–Wilk tests to assess normality. The data did not comply with normality and thus were arcsine square root transformed (emergence data) or log10 transformed before subjecting them to two-way ANOVAs and Tukey’s HSD post hoc tests. The statistical analyses were performed using R studio version 3.4.3 [52], with the significance level set at *p* < 0.05 (α of 0.05).

Bioinformatic analyses were performed at the Canadian Centre for Computational Genomics (C3G). Illumina CASAVA pipeline was used for base calling. Trimming and clipping of adapters were performed using Trimmomatic software [53]. Read sets aligned to the honey bee reference genome, *Apis mellifera* (v. Amel_4.5) [54], using STAR [55]. The RNAseq fragment counts were normalized based on their length. Aligned RNAseq reads were assembled into transcripts, and Cufflinks was used to calculate their abundance in fragments per kilobase of exon per million fragments mapped (FPKM) [56]. For quality control, a pairwise sample correlation analysis was performed to detect transcript expression consistency between samples. Differential gene expression analysis was performed using DESeq R Bioconductor package [57], and edge R Bioconductor package [58] based on the raw read counts generated by HTSeq [59]. Transcript expression levels and test for significant differences (*p* < 0.05) were calculated with Cuffdiff [56] based on the FPKM values calculated by Cufflinks [56]. To compare the fold change of the DEGs of different pairwise comparisons, the data were subjected to the Kruskal-Wallis test and the Conover–Iman procedure, as it did not comply with normality based on the Shapiro–Wilk test ( *p* < 0.05, α of 0.05).

The association of biological pathways with the differentially expressed genes (DEGs) was determined by the KASS-KEGG automatic annotation server [60] with the Kyoto Encyclopaedia of Genes and Genomes (KEGG) [61] by inputting the nucleotide sequences of the DEGs. Venn diagrams were created with the DEGs from the pairwise comparison using the Bioinformatics and Evolutionary Genomics [62].

## 3. Results

### 3.1. Effect of Clothianidin and/or V. destructor on Bee Emergence and Weight

Exposure of larvae to clothianidin did not affect the proportion of bees that emerged at any dose (F_(3,16)_ = 0.422, *p* = 0.740), but *V. destructor* significantly decreased the proportion of emerged bees, with or without clothianidin exposure (F_(1,16)_ = 55.65, *p* < 0.0001) (Table 1). No interaction between exposure to clothianidin and *V. destructor* parasitism was observed (F_(3,16)_ = 0.92, *p* = 0.916). Thus, the only factor linked to a decrease in bee emergence was *V. destructor* parasitism.

Larvae exposed to 1.33 ng of clothianidin were significantly lighter at emergence (117.76 ± 1.43 mg) than bees exposed to 0.13 ng of clothianidin (122.891 ± 1.09 mg), but none of the clothianidin treatments applied alone differed for weight from the control (F_(3,808)_ = 2.75, *p* = 0.042) (Table 2). However, *V. destructor* significantly reduced the weight of newly emerged bees compared to the control or any dose of clothianidin alone (F_(1,808)_ = 149.99, *p* < 0.0001). Bees exposed to the combined stressors had a significantly lower weight compared to bees exposed to 0.67 ng of clothianidin alone (113.67 ± 1.05 and 120.81 ± 1.16 mg, respectively) but significantly higher weight compared to bees only parasitized by *V. destructor* (111.82 ± 1.09 mg). An interaction between clothianidin and *V. destructor* was detected (F_(3,808)_ = 5.34, *p* = 0.001).

### 3.2. Effect of Clothianidin and/or V. destructor on Cellular Immunity

Larvae exposed to the lowest dose of clothianidin (0.13 ng/bee) had a significantly higher number of haemocytes after emergence compared to the control (23,645.8 ± 1695.45 and 16,078 ± 920, *p* < 0.05), although the medium and highest doses of clothianidin did not affect haemocyte counts of the newly emerged bees (*p* > 0.05; Table 3). *V. destructor* parasitism of larvae resulted in significantly lower number of haemocytes after emergence, with or without clothianidin (F_(1,226)_ = 59.595, *p* < 0.0001). No interaction between clothianidin exposure and *V. destructor* parasitism was observed on the number of haemocytes in newly emerged bees.

### 3.3. Effect of Clothianidin and/or V. destructor on DWV Levels

**Figure 1:**
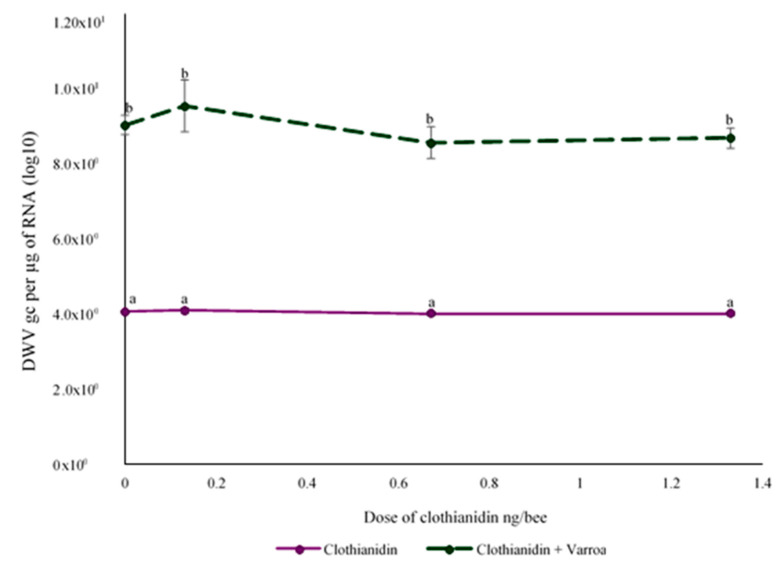
Mean deformed wing virus (DWV) genome copies (GCs) per µg of RNA (± S.E.) of emerged bees that were exposed to clothianidin and/or *V. destructor* during the larval stage. Different letters above the bars indicate significant differences based on a two-way ANOVA and Tukey’s HSD tests. Log10-transformed data are presented.

Exposure to clothianidin during the larval stage did not have a significant effect on DWV levels in newly emerged bees (F_(3,64)_ = 1.021, *p* < 0.390) (Figure 1). However, the newly emerged bees that were parasitized as larvae by *V. destructor* had 2.23 × 105 more DWVgc per µg of RNA than non-infested larvae, which was significant (F_(1,64)_ = 430.670, *p* ≤ 0.0001). There was no interaction between the two factors (F_(3,64)_ = 1.012, *p* = 0.556; Figure 1). Additionally, DWV levels in bees that emerged from *V. destructor*-parasitized larvae without clothianidin were not significantly different to those of bees that were parasitized and also exposed to clothianidin at any dose tested (*p* > 0.05). Therefore, *V. destructor* was the main factor contributing to DWV levels.

### 3.4. Comparisons of Up and Down-Regulated DEGs

**Figure 2:**
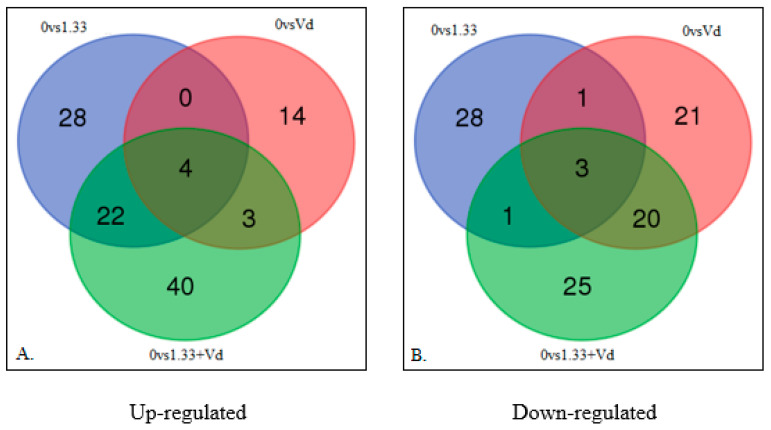
Venn diagram showing number of DEGs based on the differential gene expression analysis, and the genes in common between the pairwise comparisons of 0 ng of clothianidin vs. 0.1.33 ng of clothianidin (0 vs. 0.1.33), 0 ng vs. 0 ng plus *V. destructor* (0 vs. Vd) and 0 ng vs. 1.33 ng of clothianidin plus *V. destructor* (0 vs. 1.33+Vd). (**A**) Venn diagram showing the number of up-regulated DEGs (**B**) Venn diagram showing the number of down-regulated DEGs.

For determining the number of DEGs, 18,989,563 reads for the non-treated control, 20,215,459 reads for 1.33 ng of clothianidin alone, 15,347,021 reads for *V. destructor* alone, and 19,761,070 reads for 1.33 ng of clothianidin plus *V. destructor* were obtained. Pairwise comparisons for up-regulated DEGs between the control and 1.33 ng/bee of clothianidin, *V. destructor* and the combination identified 54, 21 and 69 DEGs, respectively (Figure 2A, Table S1). Thus, clothianidin alone up-regulated approximately double the number of DEGs than *V. destructor* alone, but the broadest impact appeared to be caused by the combined stressors with the highest number of up-regulated DEGs, indicating a possible interaction between the stressors. There were shared up-regulated DEGs between treatments, which was higher for clothianidin with the combined stressors than for *V. destructor* with the combined stressors or clothianidin alone with *V. destructor* alone (26 versus 7 or 4 DEGs, respectively). Pairwise comparisons for down-regulated DEGs showed 33, 45 and 49 for clothianidin, *V. destructor* and the combination compared to the control, respectively (Figure 2B, Table S1). Thus, *V. destructor* alone down-regulated more DEGs than clothianidin alone, but the greatest number occurred with the combined stressors, once again indicating a possible interaction between the stressors. The number of shared down-regulated DEGs from the pairwise comparisons was higher for *V. destructor* with the combined stressors than for clothianidin with the combined stressors or clothianidin alone with *V. destructor* alone (23 versus 4 or 4 DEGs, respectively). Thus, the effect of clothianidin was greater than *V. destructor* for up-regulation, but the reverse was true for down-regulation, implying a more stimulatory effect of clothianidin and a more suppressive effect of *V. destructor* based on DEG numbers. Having relatively more DEGs with a treatment was also reflected in having more shared DEGs with the combination of clothianidin and *V. destructor*, implying that it was the up-regulatory effects of clothianidin and the down-regulatory effects of *V. destructor* that were more dominant when the two stressors were combined.

There were also differences in the magnitude of the DEG up- or down-regulation (*Chi^2^* = 11.07, *p* < 0.0001, *df* = 5). For up-regulated DEGs with clothianidin, the average fold change was 1.49 ± 0.16, which was not significantly different from that observed with *V. destructor* (1.56 ± 0.11, *p* > 0.05), but was significantly lower than the fold change of clothianidin plus *V. destructor* (1.64 ± 0.82, *p* < 0.05). The average fold change for down-regulated DEGs with clothianidin (1.93 ± 0.24) was significantly lower than that observed with *V. destructor* (2.56 ± 0.30) and clothianidin plus *V. destructor* (2.60 ± 0.31, *p* < 0.05). In addition, the average fold change for down-regulation of each treatment was significantly greater than the fold change for up-regulation (*p* < 0.0001), indicating that the average down-regulatory impact was greater.

### 3.5. KEGG Analysis

There were 80 KEGG pathways linked with the up-regulated DEGs (Table S2). Among them, 11 were associated only with clothianidin, including the insulin signaling pathway and insulin resistance (Table S2), 25 were associated only with *V. destructor*, including leukocyte transendothelial migration and phagosome (Table S3), and 33 were associated only with the combined stressors, including metabolism of xenobiotics by cytochrome P450, and fat digestion and absorption (Table S4). For the up-regulated DEGs shared between treatments, the seven KEGG pathways shared between clothianidin and the combined stressors were all related to metabolic pathways, like carbon metabolism and biosynthesis of secondary metabolites, the two KEGG pathways shared between *V. destructor* and the combined stressors were Salmonella infection and proteoglycans in cancer, no KEGG pathways were shared between clothianidin and *V. destructor*, and only one KEGG pathway was shared by each stressor alone and the combined stressors, which was Huntington’s disease.

For the down-regulated DEGs, 56 were associated with KEGG pathways. There were 12 pathways linked to DEGs only with clothianidin, such as nicotine addiction and glutamatergic synapse (Table S5), no pathways were linked only to *V. destructor* parasitism (Table S6), and 33 pathways linked only to the combined stressors, from which 62% were metabolic terms, and 8% were terms associated with drug metabolism (Table S7). For the down-regulated DEGs shared between treatments, three KEGG pathways were shared between clothianidin and the combined stressors that were pancreatic secretion, microbial metabolism in diverse environments, and carbon metabolism, six KEGG pathways were shared between *V. destructor* and the combined stressors that were metabolic pathways, glycine, serine and threonine metabolism, biosynthesis of antibiotics, purine metabolism, and biosynthesis of secondary metabolites, and no KEGG pathways were shared between clothianidin and *V. destructor* or by each stressor alone and the combined stressors.

## 4. Discussion

The main factor associated with mortality at emergence was *V. destructor* parasitism, as sublethal exposure to clothianidin did not affect the proportion of bees that emerged and no interaction between the two stressors was observed. The feeding behavior of *V. destructor* could be related to the high mortality rate. *V. destructor* pierces the cuticle of larvae or pupae and then feeds on their fat body [63]. Hence, the intake of fat body tissue by the parasite could have negatively impacted the development of the bees sufficiently to result in death of some of the brood. Further, the reduced proportion of bee emergence could be due to *V. destructor* salival secretions damaging haemocytes and preventing the formation of haemocyte aggregates, leading to an aberrant wound healing process [64]. Additionally, the effect of viral infections vectored by the mite could increase mortality by immunosuppressing humoral responses [37,41,65]. An increase in larval mortality due to *V. destructor* parasitism has been previously reported, and our findings closely match those studies [34,66,67].

A reduction in weight was observed in newly emerged bees exposed to the highest dose of clothianidin alone and *V. destructor* alone, and an interaction between clothianidin and *V. destructor* was observed. Weight at emergence is an indicator of normal physiological functioning, and thus a reduction in weight is evidence of stress during larval development. The loss of weight caused by the highest dose of clothianidin in this study agrees with previous findings on the effect of neonicotinoid insecticides in other insects, like reduced weight in *Aphis gossypii* after imidacloprid exposure [68]. The reduction in weight by *V. destructor* parasitism in newly emerged bees also confirms previous findings, which reported a significant loss of body weight in newly emerged bees [33,69]. Clothianidin and *V. destructor* have been found to reduce the body weight of bees and other insects, but this is the first study showing an interaction with the combination of clothianidin and *V. destructor* on weight at emergence of bees. However, the effects were not additive, as the weight at emergence was not lower with the combination of the medium dose of clothianidin plus *V. destructor* than with the mite alone. Further, it appeared that the interaction depended on the dose, and hence, studies using a wider range of doses could help understand the dose effect in combination with the parasite on weight at emergence.

The concentration of haemocytes in the haemolymph of newly emerged bees increased significantly with the lowest lethal dose of clothianidin and decreased significantly with *V. destructor* parasitism, but no interaction between the two stressors was noted. The decreased number of haemocytes due to *V. destructor* could be due to the mite’s saliva, which reduces the viability of haemocytes [64], but could also be due to the recruitment of haemocytes towards the wound site, leading to a decrease in the number of circulating haemocytes [27]. Koleoglu et al. [38] and Salem et al. [70] reported a decrease in haemocyte counts in parasitized adult worker bees and parasitized adult drones and larvae, respectively. Similar to this study, Amdam et al. [71] showed that larvae parasitized by *V. destructor* resulted in newly emerged adults with significantly lower haemocyte counts. Reductions were not observed in the number of haemocytes in adult bees treated for 24 h with sublethal doses of clothianidin using similar sublethal doses as in this study, but much higher doses (50–20 ng/µL which was 200–500-fold higher than the 0.13 ng/µL clothianidin in this study) resulted in reduced haemocytes, which was also observed with those higher doses of thiacloprid and imidacloprid [72]. Surprisingly, the lowest lethal dose of clothianidin increased the number of haemocytes. This increase could be explained by hormesis, in which a sublethal dose of a toxin stimulates a potentially beneficial response [73]. Hormesis has been observed after exposure to sublethal doses of the insecticide deltamethrin in *Sitophilus zeamais*, which caused an increase in population growth [74]. Further, after sublethal exposure to a biopesticide (*Bacillus subtilis*), *Bombus impatiens* showed an hormetic response by a significant increase in drone production [75]. In this study, haemocyte counts were evaluated 13 days after the last exposure to clothianidin, indicating a long-term effect of the insecticide on cellular immunity. The mechanisms behind the possible hormetic response on cellular immunity in bees by sublethal exposure to insecticides deserves further investigation.

DWV levels in newly emerged bees for *V. destructor*-parasitized larvae were significantly higher than in control bees, but the clothianidin only treatment had no effect, and there was no evidence of an interaction between clothianidin and *V. destructor* on DWV levels. There are other reports of DWV increasing with *V. destructor* parasitism of larvae [34,76]. It has been proposed that this is related to the detrimental effect of *V. destructor* on the bees’ immune system [36,37,38], as well as the role of *V. destructor* as a biological vector and replicator of the virus [77,78]. A possible immunosuppressive effect of clothianidin increasing viral levels was not found in this study, although it has been previously reported in exposed adult bees [22], suggesting that bees exposed to a neonicotinoid insecticide during the larval stage do not increase their susceptibility to DWV replication the same way as when adult bees are exposed.

The analysis on the number of DEGs may have revealed some of the mechanisms related to the effects observed for emergence, weight at emergence, haemocyte concentration and DWV levels with the stressors alone or in combination. For clothianidin alone, up-regulated DEGs were associated with central metabolic pathways, such as carbon metabolism and biosynthesis of amino acids, whereas down-regulated DEGs were associated with glutamatergic synapse and neuroactive ligand–receptor interaction. This may be a result of stimulation of metabolism and a moderate activation of nAChRs, causing nervous stimulation [79]. Similar results were previously obtained showing that exposure of adult bees to thiamethoxam resulted in DEGs linked to pathways related to metabolism, such as tyrosine metabolism and pentose and glucoronate interconversion and drug metabolism [80]. Further, DEGs in brains of bees were found related to metabolic processes, including starch and sucrose metabolism, following sublethal doses of clothianidin and imidacloprid [81], which is similar to the findings of this study. While clothianidin alone did not significantly affect the factors tested in this study, the detection of DEGs showed that the metabolism of the bees was likely altered but perhaps not sufficiently to affect the factors tested.

For *V. destructor* alone, parasitism negatively affected emergence, weight at emergence, haemocyte counts and DWV levels. In this study, *V. destructor* parasitism resulted in up-regulated DEGs, including actin clone 205-like and peptidoglycan recognition protein 1, related to biological pathways associated with cellular immunity, such as phagosome, platelet activation and leukocyte trans-endothelial migration. It also resulted in down-regulated DEGs, including abaecin and hymenoptaecin, associated with humoral immune responses, such as Salmonella infection and streptomycin synthesis, that were also conserved when combined with clothianidin. This could be related to the effect of *V. destructor* on immune defense mechanisms, perhaps allowing pathogens like DWV to increase more, eventually contributing to reduced emergence and weight at emergence.

There was an interaction observed between the stressors for reduced weight of the newly emerged bees. The stressors alone up-regulated and down-regulated fewer DEGs compared to the combined stressors, indicating a possible additive and subtractive effect on the metabolism of the bee when combined. It was also notable that there were more shared DEGs between clothianidin and the combined stressors for up-regulated DEGS, but more shared DEGs between *V. destructor* and the combined stressors for down-regulated DEGS, showing that the combination of the stressors appeared to reflect more dominant effects of clothianidin for up-regulation and *V. destructor* for down-regulation when combined. However, there were unique up-regulated DEGs with the combined stressors, including cytochrome P450 304a1 and cytochrome P450 6a2, linked to KEGG terms for drug metabolism and metabolism of xenobiotics, indicating that clothianidin and *V. destructor* can interact causing new effects not observed with the stressors alone. These effects appeared to be primarily related to the detoxification abilities of the bee’s metabolism. The combined stressors also showed shared effects with other stressors with up-regulated DEGs, such as phospholipase A2 and apyrase, which are associated with KEGG terms related to energy metabolism, particularly carbohydrates and fats. The combined stressors also down-regulated DEGS, such as ornithine aminotransferase and glycine *N*-methyltransferase, associated with KEGG terms related to amino acid and energy metabolism, showing that the interaction between the mite and clothianidin could impact multiple key biological functions important for survival. These changes could be related to reduced weight at emergence due to the energetic costs associated with detoxification processes and altered energy metabolism in the developing larvae.

In this study, *V. destructor* was the main stressor associated with negative effects on survivorship, weight, haemocyte counts, DWV levels and biological pathways related to immune responses, confirming a significant detrimental effect of the parasite on honey bee health. However, the combination of *V. destructor* with clothianidin had additional effects observed on bee weight and the number of DEGs compared to the stressors alone. Thus, it appears that the combined stressors are able to have long-term effects on gene regulation, potentially affecting a broad range of biological pathways, which could affect the ability of the bees to metabolize and detoxify the neurotoxin, repair tissue damage or fight off infections like DWV that are essential for development and survival. Further research on the effect of the long-term effects of combined stressors in bees exposed during the developmental stage are needed to better understand their impact on the developing honey bee in order to design strategies to prevent honey bee colony losses.

## Figures and Tables

**Table 1:** Mean proportion of emerged bees (± S.E.) exposed to sublethal doses of clothianidin (ng/bee) and/or *V. destructor*.

Treatment	Mean Proportion of Emerged Bees (± S.E.)
0 ng/bee	0.98 ± 0.0027 ^a^
0.13 ng/bee	0.96 ± 0.0069 ^a^
0.67 ng/bee	0.97 ± 0.0047 ^a^
1.33 ng/bee	0.95 ± 0.0050 ^a^
0 ng/bee + *V. destructor*	0.68 ± 0.083 ^b^
0.13 ng/bee + *V. destructor*	0.65 ± 0.12 ^b^
0.67 ng/bee + *V. destructor*	0.56 ± 0.12 ^b^
1.33 ng/bee + *V. destructor*	0.60 ± 0.10 ^b^

Different letters indicate significant differences based on a two-way ANOVA and Tukey’s HSD tests of arcsine square root-transformed data. Non-transformed data are presented.

**Table 2:** Mean weight of newly emerged bees (± S.E.) exposed to sublethal doses of clothianidin (ng/bee) and/or *V. destructor* during the larval stage.

Treatment	Mean Weight of Newly Emerged Bees (mg ± S.E.)
0 ng/bee	121.76 ±1.21 ^a,b^
0.13 ng/bee	122.89 ±1.09 ^a^
0.67 ng/bee	120.81 ± 1.16 ^a,b^
1.33 ng/bee	117.76 ± 1.42 ^b,c^
0 ng/bee + *V. destructor*	111.82 ± 1.09 ^d^
0.13 ng/bee + *V. destructor*	109.68 ± 0.94 ^e,d^
0.67 ng/bee + *V. destructor*	113.67 ± 1.05 ^c,e^
1.33 ng/bee + *V. destructor*	111.38 ± 0.92 ^e,d^

Different letters indicate significant differences based on a two-way ANOVA and Tukey’s HSD tests on log10 -transformed data. Non-transformed values are presented.

**Table 3:** Mean number of haemocytes per µL of haemolymph (± S.E.) of newly emerged bees exposed to sublethal doses of clothianidin (ng/bee) and/or *V. destructor* during the larval stage.

Treatment	Haemocytes/µL of Haemolymph (± S.E.)
0 ng/bee	16077.93 ± 920.24 ^a^
0.13 ng/bee	23645.80 ± 1695.45 ^b^
0.67 ng/bee	18058.90 ± 943.02 ^a^
1.33 ng/bee	17321.6 ± 934.07 ^a^
0 ng/bee + *V. destructor*	12445.00 ± 1209.67 ^c^
0.13 ng/bee + *V. destructor*	12571.70 ± 1105.83 ^c^
0.67 ng/bee + *V. destructor*	12962.70 ± 1307.27 ^c^
1.33 ng/bee+ *V. destructor*	11998.00 ± 1094.89 ^c^

Different letters indicate significant differences based on a two-way ANOVA and Tukey’s HSD tests on log10-transformed data. Non-transformed data are presented.

## Supplementary Materials

The following are available online at https://www.mdpi.com/2076-2607/8/6/858/s1, Table S1: Gene IDs in common between the pairwise comparisons, Table S2: KEGG pathways of up-regulated DEGs (0 vs. 1.33) [82], Table S3: KEGG pathway of up-regulated DEGs (0 vs. Vd) [82,83], Table S4: KEGG pathway of up-regulated DEGs (0 vs. 1.33+Vd) [82,83], Table S5: KEGG pathways of down-regulated DEGs (0 vs. 1.33) [82,83], Table S6: KEGG pathways of down-regulated DEGs (0 vs. Vd) [82,83], and Table S7: KEGG pathways of down-regulated DEGs (0 vs. 1.33+Vd) [82,83].
