# Supplementary material for: Interaction of Varroa destructor and Sublethal Clothianidin Doses during the Larval Stage on Subsequent Adult Honey Bee (Apis mellifera L.) Health, Cellular Immunity, Deformed Wing Virus Levels and Differential Gene Expression
Source: Microorganisms. 2020 Jun 6;8(6):858. doi: 10.3390/microorganisms8060858 (PMC7356300; doi:10.3390/microorganisms8060858)
Supplement: Supplementary file 1 [file microorganisms-08-00858-s001.pdf]

Interaction of *Varroa destructor* and Sublethal Clothianidin Doses during the Larval Stage on Subsequent Adult Honey Bee (*Apis mellifera* L.) Health, Cellular Immunity, Deformed Wing Virus Levels and Differential Gene Expression

Nuria Morfin, Paul H. Goodwin and Ernesto Guzman-Novoa

Table S1. **Gene IDs in common between the pairwise comparisons**. Gene IDs s in common between the pairwise comparisons of 0 ng of clothianidin vs 1.33 ng/µl of clothianidin (0vs1.33), 0 ng of clothianidin vs *V. destructor* (0vsVd) and 0 ng of clothianidin vs 1X10^{-2} ng/µl of clothianidin plus *V. destructor* (0vs1.33+Vd).

**Pairwise comparisons**	**Up-regulated**	**Down-regulated**
0vs1.33, 0vsVd, 0vs1.33+Vd	GB46995, GB45714, GB43710, GB50114	GB45797, GB51436, GB42668
0vs1X1.33, 0vsVd		GB46557
0vs1X1.33, 0vs1.33+Vd	GB53732, GB49509, GB51814, GB43689, GB51698, GB55213, GB44841, GB45796, GB41326, GB41912, GB55211, GB53576, GB44548, GB50977, GB46223, GB55206, GB47885, GB55209, GB55205, GB46514, GB51146, GB55212	GB42146
0vs1.33+Vd, 0vsVd	GB52278, GB40253, GB51373	GB46469, GB47318, GB50423, GB51223, GB50550, GB53798, GB50313, GB48148, GB42287, GB52100, GB50915, GB42598, GB42981, GB47546, GB44871, GB47805, GB44610, GB51306, GB50916, GB46640,
0vs1.33	GB44842, GB47362, GB43927, GB56000, GB49416, GB48881, GB41777, GB43039, GB49544, GB44710, GB47527, GB42768, GB41776, GB53041, GB52836, GB46308, GB54569, GB43789, GB47569, GB40503, GB53755, GB40608, GB56028, GB40148, GB47579, GB50262, GB54391, GB52667	GB52318, GB53641, GB46222, GB46834, GB45248, GB49887, GB48832, GB40063, GB51671, GB45696, GB52528, GB42460, GB45170, GB41839, GB49219, GB52317, GB44367, GB48510, GB48975, GB51029, GB47536, GB40038, GB50137, GB52837, GB43324, GB54419, GB55016, GB53986
0vsVd	GB50975, GB41306, GB44139, GB51089, GB55158, GB54269, GB54893, GB52910, GB42612, GB44561, GB53113, GB41311, GB44649, GB49105	GB51446, GB50477, GB52023, GB51840, GB55213, GB53369, GB43508, GB41932, GB48109, GB55211, GB50151, GB51815, GB55593, GB43247, GB47804, GB41833, GB55921, GB52184, GB40148, GB45906, GB55212
0vs1.33+Vd	GB43516, GB46427, GB43916, GB52505, GB52269, GB40285, GB41760, GB51845, GB54861, GB55143, GB43007, GB43006, GB46225, GB40624, GB48656, GB43512, GB49854, GB44070, GB55208, GB42310, GB51724, GB46444, GB47302, GB41418, GB49875, GB50629, GB47278, GB49876, GB40905, GB54997, GB44122, GB48228, GB44120, GB54260, GB52864, GB52756, GB48850, GB40074, GB43310, GB43509	GB41110, GB40566, GB45986, GB47721, GB53110, GB47696, GB42554, GB45763, GB42888, GB42310, GB48626, GB43173, GB41015, GB49441, GB54097, GB40905, GB45850, GB42597, GB51631, GB54504, GB42701, GB46612, GB41965, GB50218, GB50363

Table S2. **KEGG pathways of up-regulated DEGs (0vs1.33)**. KEGG pathways analysis of the DEGs (up-regulated) between the newly emerged bees treated with 0 ng and 1.33 ng of clothianidin during the larval stage (0vs1.33).

**Gene ID**[a]	**Gene description**[b]	**Biological pathway**[c]
GB47362	ring finger protein nhl-1	microRNAs in cancer (ko05206)
GB54569	growth arrest-specific 1	hedgehog signaling pathway (ko04340)
GB41912	oxidoreductase YrbE-like	metabolic pathway (ko01100); biosynthesis of antibiotics (ko01130); microbial metabolism in diverse environments (ko01120); microbial metabolism in diverse environments (ko001120); inositol phosphate metabolism (ko00562)
GB51814	glucose dehydrogenase	metabolic pathway (ko01100); glycine, serine and threonine metabolism (ko00260)
GB44710	L-threonine ammonia-lyase	metabolic pathway (ko01100); glycine, serine and threonine metabolism (ko00260); biosynthesis of antibiotics (ko001130); biosynthesis of amino acids (ko01230); carbon metabolism (ko01200); biosynthesis of secondary metabolites (ko01110); Huntington's disease (ko05016)
GB48881	C-1-tetrahydrofolate synthase	metabolic pathway (ko01100)
GB40503	D-3-phosphoglycerate dehydrogenase	metabolic pathway (ko01100); glycine, serine and threonine metabolism (ko00260); biosynthesis of amino acids (ko01230); carbon metabolism (ko01200); microbial metabolism in diverse environments (ko01120); biosynthesis of antibiotics (ko01130); methane metabolism (ko00680)
GB44841	methylthioribose-1-phosphate isomerase	metabolic pathway (ko01100); cysteine and methionine metabolism (ko00270)
GB54391	glycogen [starch] synthase	metabolic pathway (ko01100); glucagon signaling pathway (ko04922); AMPK signaling pathway (ko04152); insulin resistance (ko04931); insulin signaling pathway (ko04931); starch and sucrose metabolism (ko00500)

[a]Gene ID, BeeBase gene identifiers of the Honey bee genome assembly 4.5 [82,83]

[b]Gene description based on the National Center for Biotechnology Information, US National Library of Medicine Amel_4.5 [82].

[c]Bioloical pathways and (KO) identifiers from a biological pathway based on KASS search [60]

Table S3. **KEGG pathway of up-regulated DEGs (0vsVd)**. KEGG pathways analysis of the DEGs (up-regulated) between the newly emerged bees parasitized with *V. destructo*r compared to bees exposed to 0 ng of clothianidin during the larval stage (0vsVd).

**Gene ID**[a]	**Gene description**[b]	**Biological pathway**[c]
GB50114	dynein beta chain	Huntington's disease (ko05016)
GB52278	filamin like	MAPK singaling pathway (ko04010); Salmonella infection (ko05132) ; focal adhesion (ko04510); proteoglycans in cancer (ko05205)
GB41306	actin, clone 205-like	Rap1 signaling pathway (ko04015); hippo signalling pathway-fly (ko04391); phagosome (ko04145); apoptosis (ko04210); focal adhesion (ko04437); adherens junction (ko04520); tight junction (ko04530); regulation of actin cytoskeleton (ko04810); platelet activation (ko04611); leukocyte transendothelial migration (ko04670); oxytocin signalling pathway (ko04921); thyroid hormone signaling pathway (ko04919); phototransduction (ko04745); proteoglycans in cancer (ko05205); fluid shear stress and atherosclerosis (ko05418); hypertrophic cardiomyopathy (ko05410); arrythmogenic right ventricular cardiomyopathy (ko05412); dilated cardiomyopathy (ko05414); viral myocarditis (ko05414); *Vibrio cholerae* infection (ko05110); pathogenic *E. coli* infection (ko05692); *Salmonella* infection (ko05132); Shigellosis (ko05131); bacterial invasion of epithelial cells (ko05100); influenza A (ko05164)
GB44139	calmodulin-lysine N-methyltransferase	lysine degradation (ko00310)

[a]Gene ID, BeeBase gene identifiers of the Honey bee genome assembly 4.5 [82,83]

[b]Gene description based on the National Center for Biotechnology Information, US National Library of Medicine Amel_4.5 [82].

[c]Bioloical pathways and (KO) identifiers from a biological pathway based on KASS search [60]

Table S4. **KEGG pathway of up-regulated DEGs (0vs1.33+Vd)**. KEGG pathways analysis of the DEGs (up-regulated) between the newly emerged bees exposed to 1.33 ng of clothianidin plus *V. destructor* compared to bees exposed to 0 ng of clothianidin (0vs1.33+Vd).

**Gene ID**[a]	**Gene description**[b]	**Biological pathway**[c]
GB50114	dynein beta chain	Huntington's disease (ko05016)
GB41912	uncharacterized oxidoreductase YrbE-like	metabolic pathways (ko01100); microbial metabolism in diverse environments (ko01120); biosynthesis of antibiotics (ko01130); inositol phosphate metabolism (ko00562); streptomycin biosynthesis (ko00521)
GB49854	alpha-amylase	metabolic pathways (ko01100); starch and sucrose metabolism (ko0500); carbohydrate digestion and absorption (ko04973)
GB48228	phospholipase A2	metabolic pathways (ko01100); biosynthesis of secondary metabolites (ko01110); glycerophospholipid metabolism (ko00564); ether lipid metabolism (Ko00565); arachidonic acid metabolism (ko00590); linoleic acid metabolism (ko00591); alpha-linoleic acid metabolism (ko00592); ras signaling pathway (ko04014); vascular smooth muscle contraction (ko04270); pancreatic secretion (ko04972); fat digestion and absorption (ko04975)
GB52756	apyrase	metabolic pathways (ko01100); biosynthesis of secondary metabolites (ko01110); purine metabolism (ko00230); pyrimidine metabolism (ko00240); nicotinate and nicotinamide metabolism (ko00760)
GB47302	UDP-glucuronosyltransferase 1-1-like	metabolic pathways (ko01100); biosynthesis of secondary metabolites (ko01110); pentose and gluconate interconversions (ko00040); ascorbate and alderate metabolism (ko00053); steroid hormone biosynthesis (ko00140); retinol metabolism (ko00830); porphyrin and chlorophyll metabolism (ko00860); metabolism of xenobiotics by cytochrome P450 (ko00980); drug metabolism-cytochrome P450 (ko00982); drug metabolism-other enzymes (ko00983); chemical carcinogenesis (ko05204)
GB46444	serine-pyruvate aminotransferase	metabolic pathways (ko01100); biosynthesis of secondary metabolites (ko01110); microbial metabolism in diverse environments (ko01120); biosynthesis of antibiotics (ko01130); carbon metabolism (ko01200); glyoxylate and dicarboxylate metabolism (ko00630); methane metabolism (ko00680); alanine, aspartate and glutamate metabolism (ko00250); glycine, serine and threonine metabolism (ko00260); peroxisome (ko04146)
GB40074	hormone receptor-like in 38	aldosterone synthesis and secretion (ko04925)
GB51814	glucose dehydrogenase	metabolic pathways (ko01100); glycine, serine and threonine metabolism (ko00260)
GB52278	filamin-like	MAPK signaling pathway (ko04010); focal adhesion (ko04510); proteoglycans in cancer (ko05205); Salmonella infection (ko05132)
GB44841	methylthioribose-1-phosphate isomerase	metabolic pathways (ko01100); cysteine and methionine metabolism (ko00270)

[a]Gene ID, BeeBase gene identifiers of the Honey bee genome assembly 4.5 [82,83]

[b]Gene description based on the National Center for Biotechnology Information, US National Library of Medicine Amel_4.5 [82].

[c]Bioloical pathways and (KO) identifiers from a biological pathway based on KASS search [60]

Table S5. **KEGG pathways of down-regulated DEGs by (0vs1.33).** KEGG pathways analysis of the DEGs (down-regulated) between the newly emerged bees treated with 0 ng and 1.33 ng of clothianidin during the larval stage (0vs1.33).

**Gene ID**[a]	**Gene description**[b]	**Biological pathway**[c]
GB40038	bumetanide-sensitive sodium-(potassium)-chloride cotransporter-like	salivary secretion (ko04970); pancreatic secretion (ko04972); *Vibrio cholerae* infection (ko05110)
GB43324	pyruvate carboxylase	metabolic pathway (ko01100); microbial metabolism in diverse environments (ko01120); carbon metabolism (ko01200); biosynthesis of amino acids (ko01230); citrate cycle (ko00020); pyruvate metabolism (ko00620); carbon fixation pathways in prokaryotes (ko00720)
GB41839	glutamate receptor ionotropic	glutamatergic synapse (ko04724); neuroactive ligand-receptor interaction (Ko04080); cocaine (ko05030), amphetamine (ko05031), nicotine addiction (ko05033); alcoholism; cAMP signaling pathway (ko04024)

[a]Gene ID, BeeBase gene identifiers of the Honey bee genome assembly 4.5 [82,83]

[b]Gene description based on the National Center for Biotechnology Information, US National Library of Medicine Amel_4.5 [82].

[c]Bioloical pathways and (KO) identifiers from a biological pathway based on KASS search [60]

Table S6. **KEGG pathways of down-regulated DEGs (0vsVd).** KEGG pathways analysis of the DEGs (down-regulated) between the newly emerged bees parasitized with *V. destructo*r compared to bees exposed to 0 ng of clothianidin during the larval stage (0vsVd).

**Gene ID**[a]	**Gene description**[b]	**Biological pathway**[c]
GB44610	AMP deaminase 2	metabolic pathways (ko01100); biosynthesis of secondary metabolites (ko01110); biosynthesis of antibiotics (ko01130); purine metabolism (ko00230)
GB43247	alpha-glucosidase exon 2-9	metabolic pathways (ko01100); ko00052); starch and sucrose metabolism
GB44871	glycine N-methyltransferase	glycine, serine and threonine metabolism (ko00260)

[a]Gene ID, BeeBase gene identifiers of the Honey bee genome assembly 4.5 [82,83]

[b]Gene description based on the National Center for Biotechnology Information, US National Library of Medicine Amel_4.5 [82].

[c]Bioloical pathways and (KO) identifiers from a biological pathway based on KASS search [60]

Table S7. **KEGG pathways of down-regulated DEGs (0vs1.33+Vd)** KEGG pathways analysis of the DEGs (down-regulated) between the newly emerged bees exposed to 1.33 ng of clothianidin plus *V. destructor* compared to bees exposed to 0 ng of clothianidin (0vs1.33+Vd).

**Gene ID**[a]	**Gene description**[b]	**Biological pathway**[c]
GB44610	AMP deaminase 2	metabolic pathways (ko01100); biosynthesis of secondary metabolites (ko01110); biosynthesis of antibiotics (ko01130); purine metabolism (ko00230)
GB45763	tropomyosin-2-like	cardiac muscle contraction (ko04260); adrenergic signaling in cardiomyocytes (ko04261); hypertrophic cardiomyopathy (ko05410); dilated cardio myopathy (k05414)
GB54097	malvolio	lysosome (ko041421); ferroptosis (ko04216); mineral absorption (ko04978)
GB43173	chitinase	metabolic pathways (ko01100); amino sugar and nucleotide sugar metabolism (ko00520)
GB50218	ornithine aminotransferase	metabolic pathways (ko01100); biosynthesis of secondary metabolites (ko01110); biosynthesis of antibiotics (ko01130); arginine and proline metabolism (ko00330)
GB44871	glycine N-methyltransferase	glycine, serine and threonine metabolism (ko00260)

[a]Gene ID, BeeBase gene identifiers of the Honey bee genome assembly 4.5 [82,83]
[b]Gene description based on the National Center for Biotechnology Information, US National Library of Medicine Amel_4.5 [82].
[c]Bioloical pathways and (KO) identifiers from a biological pathway based on KASS search [60]
